# Hidden Diversity within *Tetralophozia filiformis* (Marchantiophyta, Anastrophyllaceae) in East Asia

**DOI:** 10.3390/plants11223121

**Published:** 2022-11-15

**Authors:** Vadim A. Bakalin, Anna A. Vilnet, Yulia D. Maltseva, Ksenia G. Klimova, Daniil A. Bakalin, Seung Se Choi

**Affiliations:** 1 Laboratory of Cryptogamic Biota, Botanical Garden-Institute FEB RAS, Makovskogo Street 142, 690024 Vladivostok, Russia; 2 Polar-Alpine Botanical Garden-Institute of the Russian Academy of Sciences, Fersmana Street, 18A, Apatity, 184209 Murmansk, Russia; 3 AXiiO Oy Company, Hämeentie, 135 A, Helsinki XR Center, 00560 Helsinki, Finland; 4 Team of National Ecosystem Survey, National Institute of Ecology, Seocheon 33657, Republic of Korea

**Keywords:** *Tetralophozia*, Anastrophyllaceae, molecular phylogenetic, integrative taxonomy, East Asia, cryptic diversity

## Abstract

*Tetralophozia filiformis* s.l. is known from a number of localities mostly in amphi-oceanic areas in Northern Hemisphere, including Atlantic Europe, amphi-Pacific Asia, South Siberia, and western North America. The newly obtained collections of this ‘species’ show strong variation in morphology of the taxon across amphi-Pacific Asia although connected by some ‘intergrading’ modifications. This implies the genetic diversity within this unit earlier recognized as a single taxon. Authors used molecular-genetic, morphological, and chorological methods to understand if the geographically correlated morphological variation also correlates with genetic differences and if it is possible to distinguish some additional taxa within the series of specimens originating from the various areas in amphi-Pacific Asia. It was found that *Tetralophozia filiformis* is a complex of at least three morphologically similar species, including one long forgotten name (*Chandonanthus pusillus*) that should be reinstated as separate species and one taxon (*Tetralophozia sibirica*) that should be described as new. *Tetralophozia filiformis* and *Chandonanthus pusillus* are lectotypified, and the new combination is provided for the latter. The three accepted taxa distinctly differ one from another in distribution patterns, preferable climate characteristics, and genetic distances, besides minor differentiations in morphology. The main morphological distinguishing features are the leaf cell size, height of undivided part in leaf lamina, and leaf dentation characteristics. Taking into account the robust correlation between the climate-based and molecular-genetic-based clusters, one more (fourth) taxon could be probably segregated from *Tetralophozia filiformis*.

## 1. Introduction

The best known species of the genus *Tetralophozia* (R.M.Schust.) Schljakov is the broadly distributed Arctic-Montane circumpolar *T. setiformis* (Ehrh.) Schljakov. Other taxa of the genus are locally distributed or rare. One of the lesser known is the predominantly East Asian *Tetralophozia filiformis* (Steph.) Urmi, originally described from Yunnan Province of China as *Chandonanthus filiformis* Steph. [1]. The distribution of *Tetralophozia filiformis* was reviewed in detail by Urmi [2], who reported this species in Europe (Spain), East Asia (Japan, Taiwan, China, North-East India, Bhutan, Malaysia), Canada, and transferred the taxon from *Chandonanthus* Mitt. to *Tetralophozia*. Urmi’s paper [2] inspired further interest to this taxon, including in Russia, where at the beginning of the new millennium, *T. filiformis* was identified for the first time in the relatively harsh oroboreal conditions of southern Siberia. It was a new report for Russian liverwort flora [3]. Following the latter, several collections of *T. filiformis* were made in south Russian Asia (Konstantinova et al. [4] and unpublished). All Siberian localities lie far enough to the north from the nearest known localities of the species in Japan and southwestern China (Sichuan, Yunnan, Taiwan Provinces), as it seemed considering the data provided by Urmi [2], supplemented by Piippo [5] and Yamada and Iwatsuki [6].

Later, Choi et al. [7] reported a taxon from the Korean Peninsula that slightly minimized the gap between the Korean-Japanese and Siberian populations of the taxon. By now, the Global Biodiversity Information Facility (GBIF) provides 234 specimen-based records of the taxon (https://www.gbif.org/ru/occurrence/search?taxon_key=2689448, accessed on 18 May 2021), all of them lying in the area described in general traits as early as by Urmi [2]. In the course of our floristic explorations in East Asia, we referred to this species several specimens collected in the areas as close as possible to the type localities of *Chandonanthus filiformis* Steph. in Yunnan Province of China and *C. pusillus* Steph. in Yamanashi Prefecture of Japan. Meanwhile, when we studied specimens in the laboratory, Japanese populations morphologically were found not to fit well with Yunnan populations, and both of them did not correspond to our specimens from Siberia. Considering the different climatic characteristics in the places of origin of the collected material, we could assume that observed differences only mirror the process of adaptation to the environments, viz. are environmentally induced and do not have any taxonomic value. However, we decided to test our observations using molecular-genetic methods. High genetic polymorphism was found in the group. Describing genetic differences and morphological, ecological, and distribution pattern variations in light of the possible speciation within the genus was the main goal of this account.

## 2. Results

### 2.1. Molecular Genetic

Seven accessions of ITS1–2, nine of *trn*L–F and a single for *trn*G-intron were produced and deposited into GenBank. The specimens from Amur Province and Khabarovsk Territory were excluded from phylogenetic estimation due to the presence of *trn*L–F data only but were used in *p*-distance calculation. The combined alignment ITS1–2 + *trn*L–F for 21 specimens consists of 1375 sites, among which 867 belong to ITS1–2 and 508 belong to *trn*L–F. The number of conserved positions in ITS1–2/*trn*L–F was 611 (70.47%)/392 (77.17%), the number of variable positions was 241 (27.80%)/106 (20.87%), and the number of parsimony-informative positions was 105 (12.11%)/37 (7.28%).

**Figure 1:**
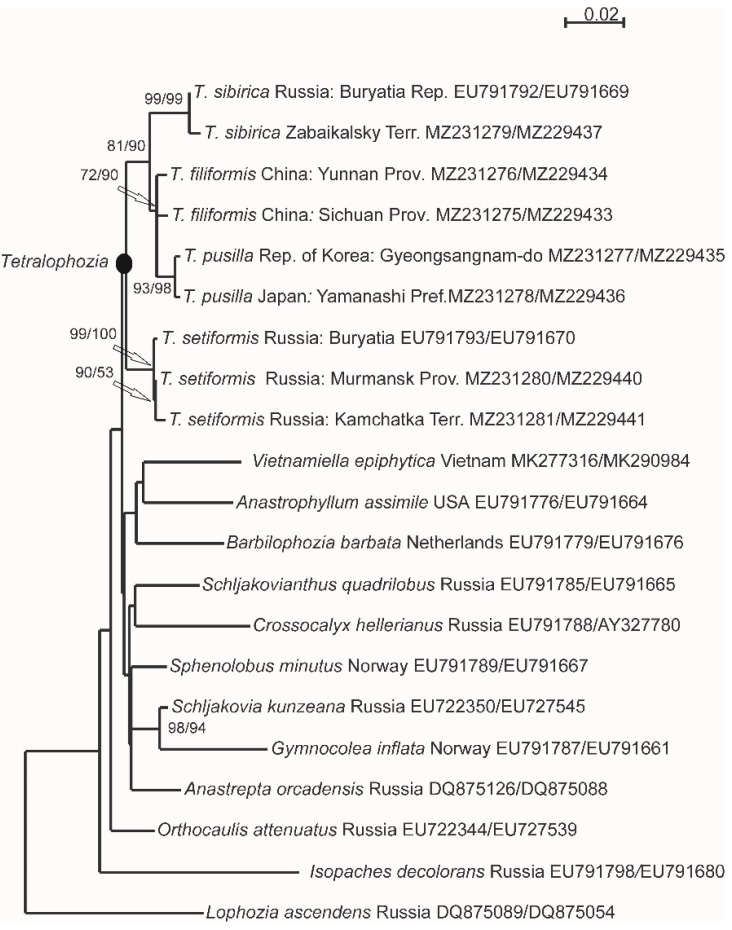
The phylogenetic tree resulted from ML analysis of the combined dataset ITS1–2 + *trn*L–F for the family Anastrophyllaceae. Bootstrap support values ≥ 50% of maximum parsimony and maximum likelihood analyses are indicated. GenBank accession numbers ITS1–2/*trn*L–F are shown.

The single most parsimonious tree with a length of 773 steps was obtained in MP analysis, consistency index—0.732394, and retention index—0.509677. The ML analysis yielded a single most likely tree (-ln *L* = 5058.463250; Figure 1). The obtained topologies are congruent among each other in the sense of relationship in the genus *Tetralophozia* but provide unsupported affinity among majority of genera in Anastrophyllaceae, as shown previously [8,9]. All specimens of *Tetralophozia* were placed in one unsupported clade. The subclade with three specimens of *T. setiformis* (bootstrap support 99% in MP, 100% in ML, or 99/100) is in a sister relationship to the subclade with the *T. filiformis* complex. Two subsequently diverged specimens of *T. filiformis* from China are sister-related (72/90) to specimens of *T. pusilla* from Japan and Korea (93/98). Both Siberian accessions compose a subclade with 99/99 support in relation (81/90) to *T. filiformis* + *T. pusilla*; these specimens we ascribe to a new species described here—*T. sibirica*.

The level of infraspecific variability did not exceed 1% in either locus in *T. setiformis*, *T. pusilla*, or *T. sibirica* and reached 1.1% in ITS1–2 in *T. filiformis* (Table 1). Within *T. filiformis* complex, *T. sibirica* is a more highly diverged species (2.5–2.7% in ITS1–2 and 1.0–1.2% in *trn*L–F) than *T. filiformis* and *T. pusilla* (1.3% in ITS1–2 and only 0.2% in *trn*L–F). *T. setiformis* was clearly distinct from all other species (2.9–3.3% in ITS1–2, 1.3–2.7% in *trn*L–F).

### 2.2. Climate Variables and the Genetic Diversification

**Figure 2:**
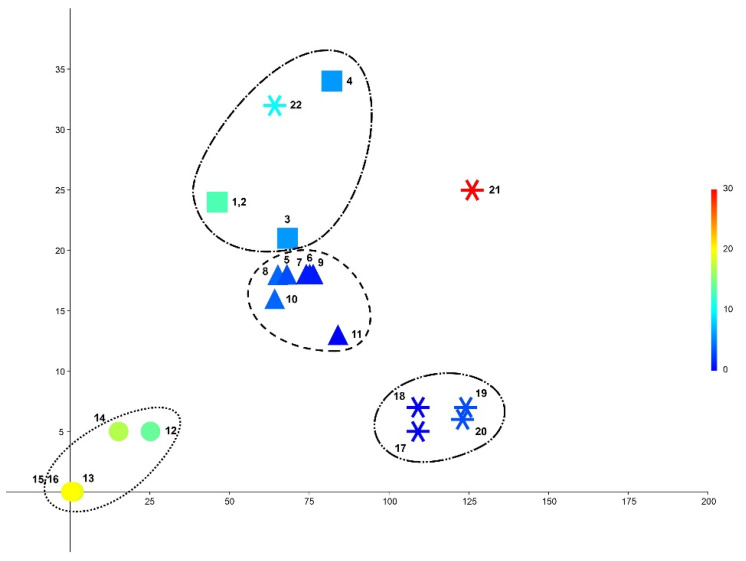
Comparison of the flora distribution in the DCA bubble chart (the third axis is the color gradient from deep blue to deep red). The taxonomical units revealed in the present account are encircled as follows: (1) dots—*Tetralophozia sibirica*, (2) dashes—*T. pusilla*, (3) dash-dotted line—*T. filiformis* s. str., (4) dash-two dots line—unknown climatic race. The specimens numbers are as in Table 3.

The bioclimate variables, as mentioned in the Section 5, were obtained for 22 geographic localities. These 22 localities corresponded to 22 specimens (both studied and unstudied by the authors). In accordance with the data obtained by molecular-genetic methods (arguing the recognition of three taxa in Asia within the *T. filiformis* complex), the specimen localities fell into four categories: (1) *T. filiformis* s. str., marked as squares, (2) *T. pusilla*, marked as triangles, (3) newly described *T. sibirica*, marked as circles and (4) specimens that we did not study but whose coordinates are precisely known, marked as snowflakes. The obtained bioclimate variables are shown in Table 2. Then, DCA was performed for a three-dimensional grid diagram. The position of each locality within a three-dimensional grid is described by formal values placed in Table 3. Then, the correlation between values of each bioclimate and values obtained in the axis of the DCA (Table 3) is placed in Table 4. Graphically, the distribution of collecting localities is presented in Figure 2. The taxonomic units revealed in the present account are encircled. Two observations are noticeable:

1. *Tetralophozia pusilla* and *T. filiformis* are closely related in the molecular-genetic respect and grow in similar climatic environments too. *Tetralophozia sibirica* grows in harsh northern environments and is genetically well different from other two taxa that also correspond distance in climate diagram.
2. Strongly geographically distanced Alaskan and Spanish populations were found to be similar in measured climate variables.

Locality 21 presumably should not be far climatically from Alaskan localities, but shows a strong difference from the climates of all other involved localities. Whether this is local aberration in the climate or regularity correlating with morphology and/or genetics was not tested here.

### 2.3. Taxonomy

As found in the molecular genetic analysis, the Korean-Japanese populations are the most closely related to the populations from Southwest China (Yunnan and Sichuan Provinces). These two groups of populations may be treated as infraspecific units, i.e., as two subspecies of the same species, especially considering the differences in the geographic patterns of the two units. However, we prefer to keep them as separate species, thus following the concept of Stephani [1], and not to create additional infraspecific taxa. Concerning the group of populations from South Siberia (and possibly the specimen from the oroboreal environments in the Russian Far East) and considering the robust genetic difference between it and Korean-Japanese plus Yunnan-Sichuan populations, the group of populations from South Siberia (and possibly the specimen from the oroboreal environments in the Russian Far East) should be treated as the distinct species.

## 3. Discussion

### 3.1. Morphology

At first glance, there is the morphological continuum connecting the largest ‘modification’ with the smallest ‘modification’ within single species. However, the comparison of the morphology with the molecular genetic data showed morphological hiatuses between revealed entities along with strictly defined regularities in the distribution. *Tetralophozia sibirica*, a species of the smallest size in the complex, has the largest cells in the leaf lobe base. The ‘intermediate’ cell size is shown by *T. filiformis*, which is characterized by the largest plants within the complex. Finally, *T. pusilla*, which is intermediate in plant size, possesses the smallest leaf cells. A similar regularity is observed in the stem cross section: the largest cells in the outer layer are in the habitually smallest *T. sibirica* (10–12 µm in diameter), while the smallest cells are in *T. pusilla* (7–10 µm in diameter) and then intermediate in size in *T. filiformis* (7–13 µm in diameter). This ‘cell size’; feature certainly possesses a quantitative nature and cannot be used alone. The further feature discriminating *T. sibirica* from other entities is the strongly thickened outer cell walls in the stem cross section (versus slightly thickened to virtually thin), while inner cells are commonly thick-walled (versus invariably thin in the remaining taxa). The trigones in the stem cross section are always convex in *T. sibirica* (versus mostly concave in two other). The same regularity is observed in the inner cells of the stem cross section.

Moreover, *T. filiformis*, the largest taxon of the group, has the thinnest cell walls in the inner part of the stem cross section and smaller (moderate in size, not large) trigones. One feature is a very speculative, although should also be mentioned. The leaf lobes in *T. pusilla* and *T. filiformis* are somewhat tuned not only to the stem apex, but also slightly so to the dorsal side to the stem, which provides to the plants the appearance somewhat similar to depauperate *Herbertus*.

The papillae are prominently coarse in *Tetralophozia filiformis* (well observable in relatively fresh specimens, less than 10 years old), while the papillae in two other taxa of the complex are slightly developed to virtually absent.

Two other features that distinguish these three taxa transform gradually in the row *T. sibirica*—*T. pusilla*—*T. filiformis*. The teeth occurring on the leaf lobes gradually become larger from *T. sibirica*, where they by 1–3 in the sinus area and developed only near the base, plus basal tooth on each lateral side of the leaf, up to 8 cells long). Then, *T. pusilla* possesses more numerous (2–4 per side in the sinus), plus lateral side basal teeth of the leaf also become larger and sometimes even branched. *Tetralophozia filiformis* shows more prominent teeth, developed in the lower 1/3–1/2 of the lobe, and the lowest of them is more than 10–13 cells long, besides each lateral side base have additional commonly curved and branched, to 20 cells long tooth. The latter taxon shows the same feature in the underleaves, whose lobes are toothed below their middles.

The undivided part in the leaf lamina also varies among the three taxa. The highest undivided part, 3–5 cells, is in the smallest species (*T. sibirica*); other taxa have larger leaves but are characterized by a lower (2–3 cells) or the same (to 6 cells) height of the undivided part. The leaves are larger in *T. filiformis* s. str. than in *T. sibirica*, which gives the impression of the much smaller undivided part in the leaf lamina in the former although in absolute value it is similar.

### 3.2. The Distribution of ‘Narrow’ Taxa of Tetralophozia filiformis Complex

The map provided by GBIF (https://www.gbif.org/ru/species/2689448, accessed on 18 May 2021) for the worldwide distribution of *Tetralophozia filiformis* s.l. is in general traits the same with the map provided almost 40 years ago by Urmi [2], with the exception of newly added reports from Russian Siberia and the Far East [3,4,10], the Republic of Korea [7] and Vietnam (the data cited in Table 5 are the new record of the species for the country). The general distribution of this species complex could be characterized as amphi-oceanic oro-boreotemperate, although the specimens in the southern extremes, including North Vietnam, were collected in orosubtropical forests. Scrutinizing the distribution of the *T. filiformis* complex in Asia, there are three taxonomical entities discussed in this account whose distribution coincides well with certain climate characteristics. *Tetralophozia filiformis* s. str. has a pronounced Sino-Himalayan distribution, extending from Indian Sikkim, West Bengal, Nepal, Bhutan to southwest China (Yunnan, Sichuan Provinces) and then to the Khoang Lien Range in northernmost Indochina (Lao Cai Province of Vietnam). The taxon is distributed in a warm monsoon climate. We do not know if the Taiwanese material belongs to the same taxon, although considering the wide penetration of many Sino-Himalayan species to Taiwan Island, *T. filiformis* s. str. can also occur there. The same may be suggested on the distribution of *T. filiformis* in Sabah (Malaysia).

*Tetralophozia pusilla*, a long-forgotten taxon, is distributed in a humid oceanic climate, and its distribution can be characterized as Japanese-Korean temperate. The northernmost collections belong to *T. sibirica*. These collections occur in a pronounced continental climate, and the type of distribution can be characterized as oroboreal Asian. 

The question remains then regarding how the populations growing outside Asia should be named. Considering that the occurrences of *Tetralophozia filiformis* s.l. in western North America are confined to cool and moderate temperate vegetation zones in the areas under oceanic climate conditions, we may assume that these American populations belong to *T. pusilla*. In contrast, the Spanish populations are unlikely to belong to the same species as the Japanese-Korean *T. pusilla*. Rather, they should belong to *T. sibirica* or another taxon that is not described yet. However, this assumption needs further study.

The DCA performed based on climate variables showed that three East Asian taxa of the complex are well differentiated by the climate conditions that confirmed the ecological requirements of all recognized taxa. Moreover, the climates in the localities of *T. filiformis* s. str. and *T. pusilla* are much closer to one another than to *T. sibirica*, which is prominently a continental climate taxon and is characterized by more robust molecular differences with the pair *T. pusilla*–*T. filiformis* s. str. Unexpectedly, the climatic localities from Spain were found in the same cluster as Alaska in the USA, which should induce further work on this species complex beyond Asia. If the climatic conditions are again found to correlate with the molecular-genetic differences, it would be quite an unusual distribution type when Pacific American specimens are related not to East Asian populations but to the temperate Atlantic.

#### Key to Tetralophozia in Amphi-Pacific Asia

1. Plants commonly more than 0.7–0.8 mm wide (depauperate modifications with commonly bilobed leaves, including ‘*f. alpina*’ are narrower), leaf middle lobe 1.5–2.2 as long as wide, leaf cuticle virtually smooth [arctic-alpine circumpolar] … *T. setiformis* *
1. Plants less than 0.7–0.8 mm wide, leaf middle lobe (2.5–)3–4 as long as wide, terminating by 1-several uniseriate cells, leaf cuticle papillose-verrucose, sometimes scarcely or coarsely so … 2.
2. Plants 0.3–0.5 mm wide, cells in the lobe base 12–30 × 12–15 µm, cuticle papillose, sometimes obscurely so, leaf basal teeth less 10 cells long [hemiboreal Asian mainland with continental climate] … *T. sibirica*
2. Plants wider, 0.6–0.75 mm wide, or almost the same size (0.4–0.6 mm wide), but then with cells in the lobe base are distinctly smaller (10–20 × 10–12 µm), leaf basal teeth less than 10 cells long or to 20 cells long [warm-temperate to tropical East Asian mainland or temperate insular-peninsular East Asia, monsoon to oceanic climates] … 3
3. Plants 0.6–0.75 mm wide, leaf undivided part 30–50 µm (2–5 cells) high, cells in the leaf lobe base 12–25 × (7–)10–12(–15) µm, cuticle distinctly papillose-verrucose, sometimes coarsely so [warm-temperate to tropical East and SE Asia] … *T. filiformis*
3. Plants 0.4–0.6 mm wide, leaf undivided part 50–90 µm (3–6 cells) high, cells in the lobe base 10–20 × 10–12 µm, cuticle distinctly papillose-verrucose, sometimes scarcely so (Korean-Japanese taxon) … *T. pusilla*

* The most recent and comprehensive treatments are in Damsholt [11], Konstantinova [3], Paton [12].

## 4. Taxonomic Treatment

### Tetralophozia sibirica Vilnet et Bakalin sp. nov.

**Figure 3:**
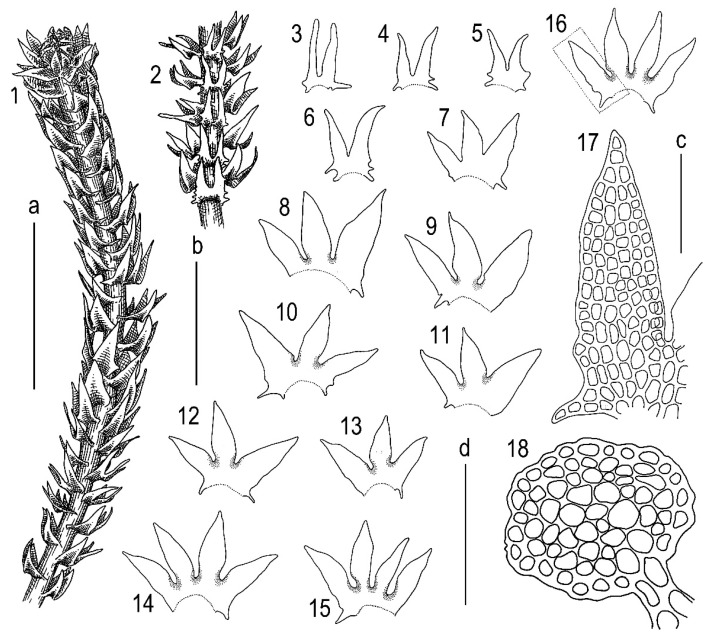
*Tetralophozia sibirica* Vilnet et Bakalin: **1**—plant habit. dorsal view. **2**—plant habit. ventral view. fragment; **3**–**6**—underleaves; **7**–**16**—leaves; **17**—leaf lobe showing cells (from 16); **18**—stem cross section; Scales: **a**—1 mm. for 1,2; **b**—500 µm. for 4–13,16; **c**—100 µm. for 17,18; **d**—500 µm. for 3,14,15. All from holotype.

**Figure 4:**
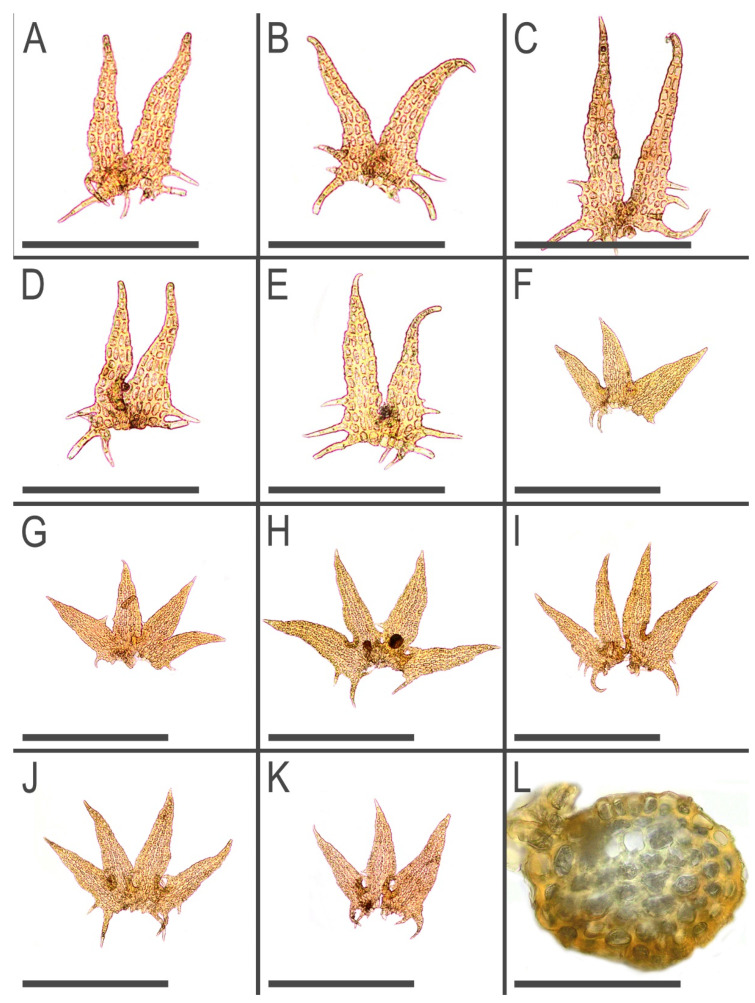
*Tetralophozia sibirica* Vilnet et Bakalin: **A**–**E**—underleaves; **F**–**K**—leaves; **L**—stem cross section; Scales: **A**–**E**—300 µm; **F**–**K**—500 µm; **L**—100 µm. All from holotype.

**Figure 5:**
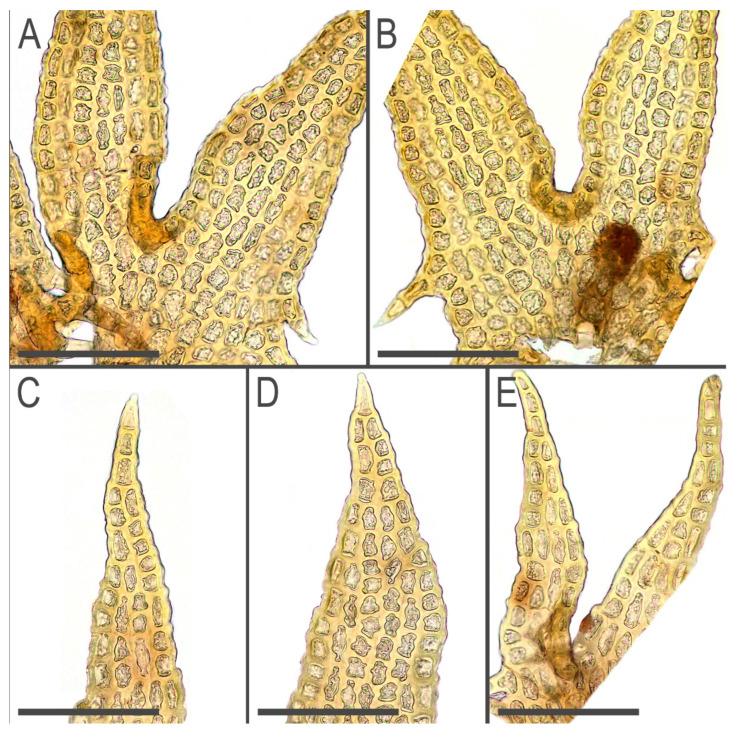
*Tetralophozia sibirica* Vilnet et Bakalin: **A**,**B**—leaf sinus area; **C**–**E**—leaf lobe apex. Scales: **A**–**E**—100 µm. All from holotype.

Description. Plants brown to yellowish brown in herbarium, when fresh commonly with green-brownish shoot apices, ascending, never distinctly creeping or erect, forming very loose mats, commonly with admixture of some pleurocarpous mosses, 10–20 mm long and 0.3–0.5 mm wide, shoots look loosely vermiform. Rhizoids virtually absent to sparse, in short brownish obliquely spreading fascicles, 250–400 µm long, originating from the stem near underleaf bases. Branching sparse, terminal, *Frullania*-type (looking as dichotomous); stem cross section slightly transversely elliptic, 100–110 × 120–130 µm, with loosely defined cortex, external wall distinctly striolate, outer cells with thick walls, irregular in shape, ca. 10–12 µm in diameter, inner cells 10–20 µm in diameter, with thickened to thin walls and large, mostly convex trigones. Leaves transversely inserted, mainly subimbricate or (rarely, in weaker shoots only) obliquely spreading; subimbricate leaves distinctly concave, with lower 1/4–1/3 erect spreading, then suddenly curved and lobes subparallel to the stem; (3–)4-lobed, lobes subequal (although weak plants have predominantly 3-lobed leaves, with dorsal lobe larger), (250–)400–450 µm long and (at the level of leaf lamina) (200–)250–300 µm wide, undivided part 50–90 µm (4–5 cells) high, lobe apices prominently acuminate, with straight axis and 1–3-celled uniseriate ends, sinus strongly recurved near its base, each lobe with (1–)2(–3) acute 1–3-celled teeth near base, besides the lateral sides of the leaf have one more additional, commonly curved, to 10 cells long teeth. Underleaves bilobed, 250–350 µm long, 120–150 µm wide (at the level of lamina), undivided part 30–50 µm (2–3 cells) high, lobes prominently acuminate with straight or distinctly curved axes and 2–4-celled uniseriate ends, sinus margin recurved in the base, lobe bases in the sinus with 2–4 teeth, while underleaf side bases with 3–5 teeth, with the basal tooth largest. Cells in the lobe base 12–30 × 12–15 µm, oblong to nearly isodiameteric, walls strongly vermiculately thickened, with prominent, large and convex trigones; cuticle papillose, sometimes obscurely so. Generative structures unknown. (Figure 3, Figure 4 and Figure 5).

Holotype: Russia, Buryatia Republic, Khamar-Daban Range, Anosovka River Valley, Levaya Anosovka River Middle course; small narrow and wet canyon with waterfall; shady moist side of the stone (51.42925N 105.040583E), 780 m a.s.l., N.A. Konstantinova 13-24-01, 04 Aug. 2001 (KPABG102424, duplicate in VBGI). Other specimens examined are in Table 5.

*Tetralophozia filiformis* (Steph.) Urmi. J. Bryol. 12 (3): 394. 1983.

Basionym: *Chandonanthus filiformis* Steph., Sp. Hepat. [1] 3: 644.

Lectotype (selected here): Ma Cul Chan, Delavay s.n. (G00112121/3698!)

Note on the lectotypification: Franz Stephani (with very limited exceptions) did not designate holotypes in the materials he studied. Moreover, the vast majority of the collections he studied were sent to him on loan and were returned to the sender after Stephani separated a small part of the specimen for his own herbarium in Leipzig (now is in G). Therefore, at least two type specimens for each taxon are presumed to exist in the majority of cases, and the formal lectotype should be designated. A discussion on this issue, including also the questionable lectotypification of Stephani’s taxa by Bonner, is provided by Engel and Merrill [13] with the corresponding references on this issue. This is why the vast majority of taxa described by Stephani should be lectotypified.

**Figure 6:**
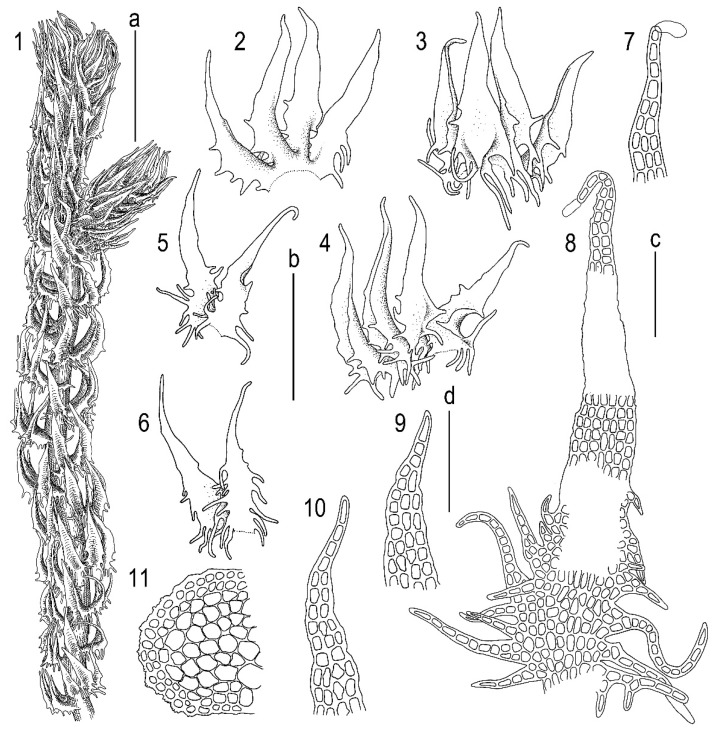
*Tetralophozia filiformis* (Steph.) Urmi: **1**—plant habit. ventral view; **2**–**4**—leaves; **5**,**6**—underleaves; **7**—underleaf lobe apex; **8**—leaf lobe; **9**,**10**—leaf lobe apex; **11**—stem cross section. fragment; Scales: **a**—1 mm. for 1; **b**—500 µm. for 2–6; **c**—100 µm. for 8; **d**—100 µm. for 7,9–11. All from China-40-16-17 (VBGI).

**Figure 7:**
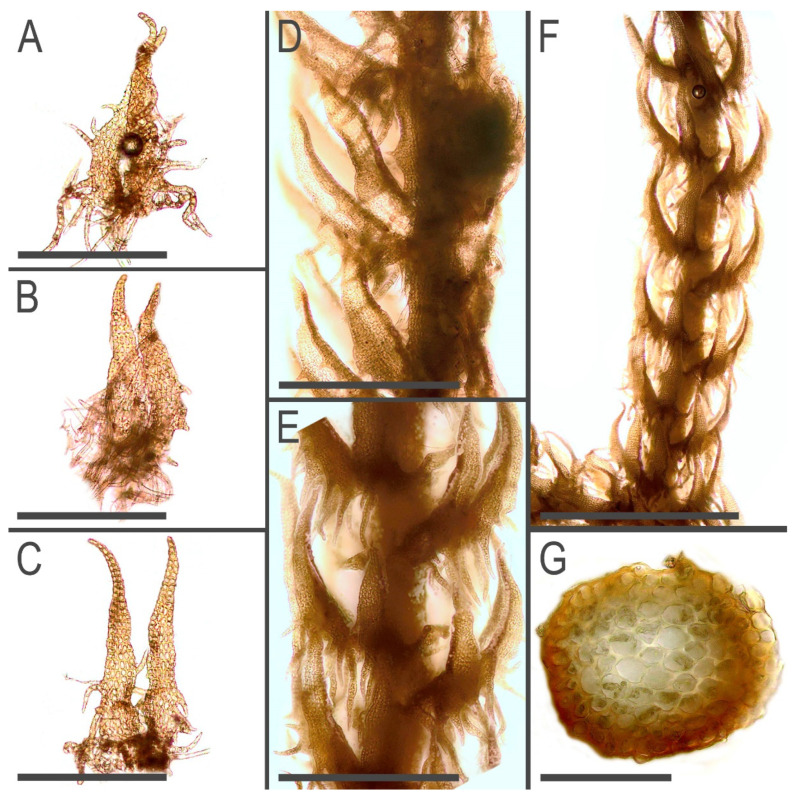
*Tetralophozia filiformis* (Steph.) Urmi: **A**–**C**—underleaves; **D**—plant habit. fragment. lateral view; **E**,**F**—plant habit. fragment dorsal view; **G**—stem cross section. Scales: **A**–**C**—300 µm; **D**,**E**—500 µm; **F**—1 mm; **G**—100 µm. All from C-39-1-17 (VBGI).

**Figure 8:**
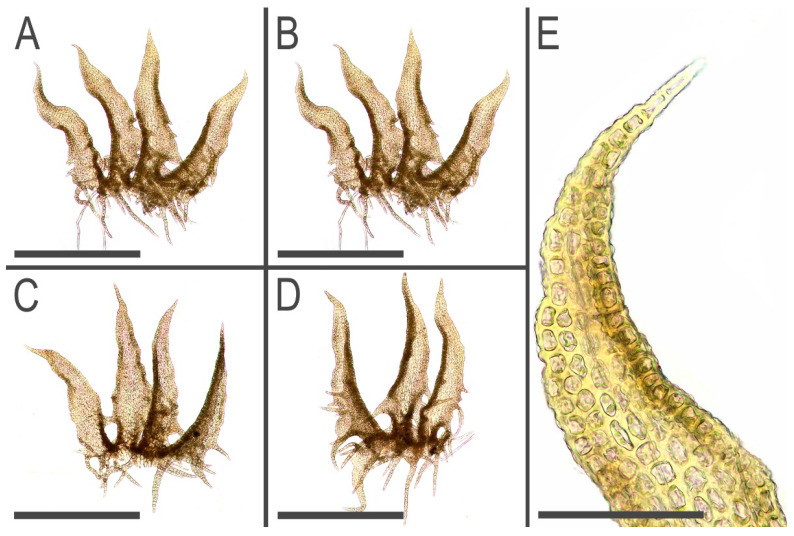
*Tetralophozia filiformis* (Steph.) Urmi: **A**–**D**—leaves; **E**—leaf lobe apex. Scales: **A**–**D**—500 µm; **E**—100 µm. All from C-39-1-17 (VBGI).

**Figure 9:**
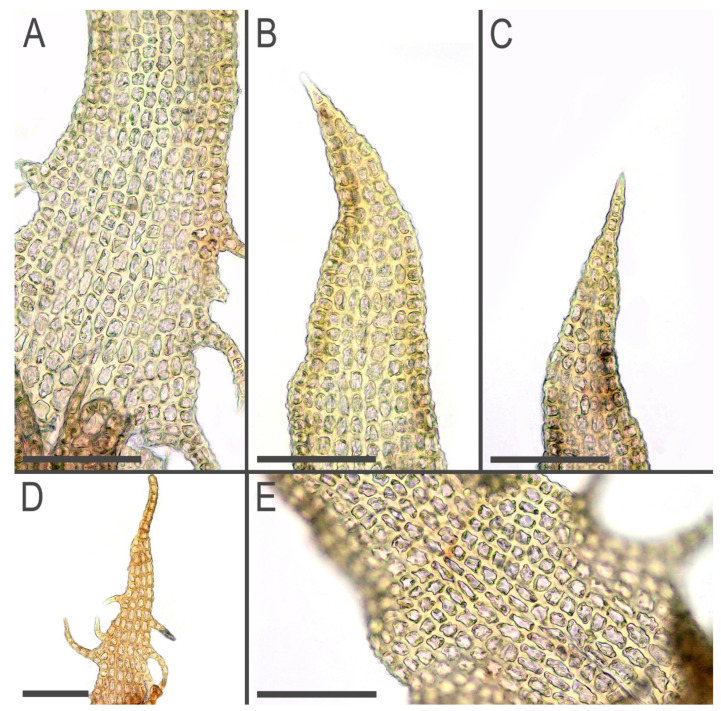
*Tetralophozia filiformis* (Steph.) Urmi: **A**,**E**—leaf lobe bases; **B**,**C**—leaf lobe apex cells; **D**—underleaf lobe apex. Scales: **A**–**E**—100 µm. All from C-39-1-17 (VBGI).

**Figure 10:**
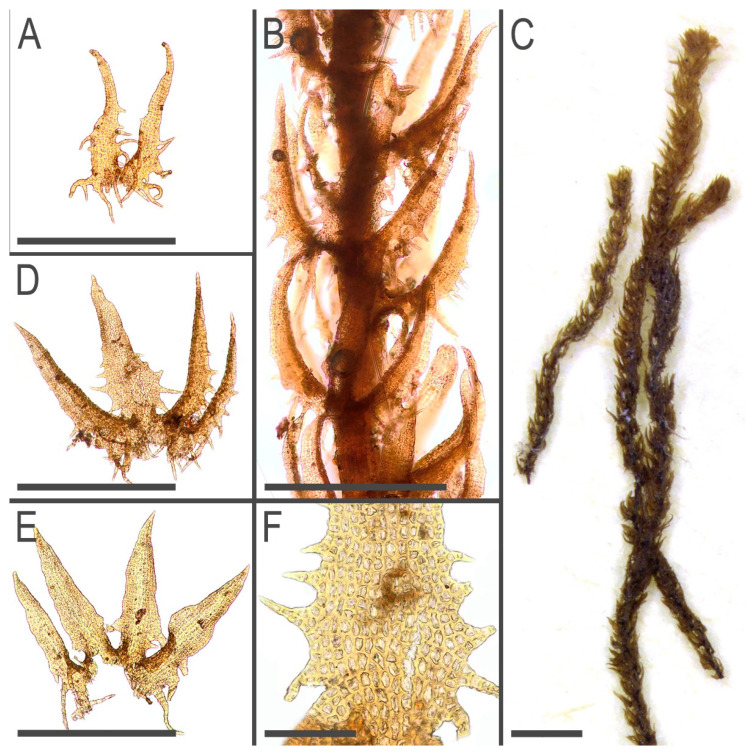
*Tetralophozia filiformis* (Steph.) Urmi: **A**—underleaf; **B**—plant habit, fragment, lateral view; **C**—plant habit; **D**,**E**—leaves; **F**—cells in leaf lobe base. Scales: **A**,**B**,**D**,**E**—500 µm; **F**—100 µm; **C**—1 mm. All from G00112121.

Description (based on specimens examined placed in the Table 5). Plants brown to yellowish brown in herbarium, when fresh commonly with green-brownish shoot apices, ascending, never distinctly creeping or erect, forming very loose mats, commonly with admixture of some pleurocarpous mosses, 15–50 mm long and 0.6–0.75 mm wide (dry plants 0.4–0.6 mm), shoots look loosely vermiform, leaves commonly obscurely turned to the dorsal side. Rhizoids virtually absent to sparse, 400–600 µm long, grayish to nearly colorless, soft, undulate, obliquely spreading, separated or in unclear fascicles, originating from the stem near underleaf bases. Branching sparse, terminal, *Frullania*-type (superficially looking as dichotomous), rarely ventral intercalary; stem cross section slightly transversely elliptic with smooth external wall, 130–150 × 160–180 µm, with loosely defined cortex, outer cells with thick walls, irregular in shape, ca. 7–13 µm in diameter, inner cells to 25 µm in diameter, with thin walls and large to moderate in size, concave trigones. Leaves transversely inserted, mainly subimbricate or (rarely, in weaker shoots only) obliquely spreading, somewhat turned dorsally; subimbricate leaves distinctly concave, with lower 1/4–1/3 erect spreading, then suddenly curved and going subparallel to the stem; (3–)4-lobed, lobes subequal (although weak plants have predominantly 3-lobed leaves, with dorsal lobe larger), 600–800 µm long and (at the level of leaf lamina) 250–350 µm wide, undivided part 30–50 µm (2–5 cells) high, lobe apices prominently acuminate, with straight axis and 2–4(–5)-celled uniseriate ends, sinus strongly recurved in the base, each lobe with 2–4 acute 3–12-celled teeth in lower 1/3–1/2, besides each lateral side of the leaf have one more additional, commonly curved and branched, to 20 cells long tooth. Underleaves bilobed, 400–500 µm long, 150–250 µm wide (at the level of lamina), undivided part 20–40 µm (2–3 cells) high, lobes prominently acuminate with straight or distinctly recurved axes and 3–6-celled uniseriate ends, if uniseriate end shorter then the lobe ends by biseriate end to 8–10 cell-pairs long, sinus margin recurved in the base, lobe lower half in the sinus side with 2–4 teeth, leaf lateral bases with 3–5 teeth, the basal tooth largest. Cells in the lobe base 12–25 × (7–)10–12(–15) µm, oblong to nearly isodiametric, walls strongly vermiculately thickened, with prominent, large, and convex trigones; cuticle distinctly papillose-verrucose, sometimes coarsely so (Generative structures in molecularly studied specimens are absent) (Figure 6, Figure 7, Figure 8, Figure 9 and Figure 10).

Comment. The type specimen of the taxon is somewhat smaller than other specimens from Yunnan and Sichuan. It has shorter but the same in width leaves and somewhat wider underleaves (although of the same length as other materials). Moreover, the plants in the lectotype are slightly crumbled, which gives the impression of smaller and shorter plants than commonly in the species. The old, dried herbarium specimens commonly have a less pronounced leaf surface armature, including papillae. The latter may be the consequence of the partial collapsing of the cells and indenting of surface elements inside. The latter is the reason the cuticle elements are not as coarse as in relatively fresh material.

*Tetralophozia pusilla* (Steph.) Bakalin et Vilnet comb. nov.

Basionym: *Chandonanthus pusillus* Steph., Sp. Hepat. [1] 3: 645.

Lectotype (selected here): Komagadake Mt., Kai, No. 35, Aug. 1903 Coll. K. Tamura G00283305/11030! Stephani [1] wrote the collector name as Yoshinaga, although the collection was only sent to Stephani by Yoshinaga (one of his Japanese correspondents), while the label distinctly indicates the collector name as K. Tamura.

**Figure 11:**
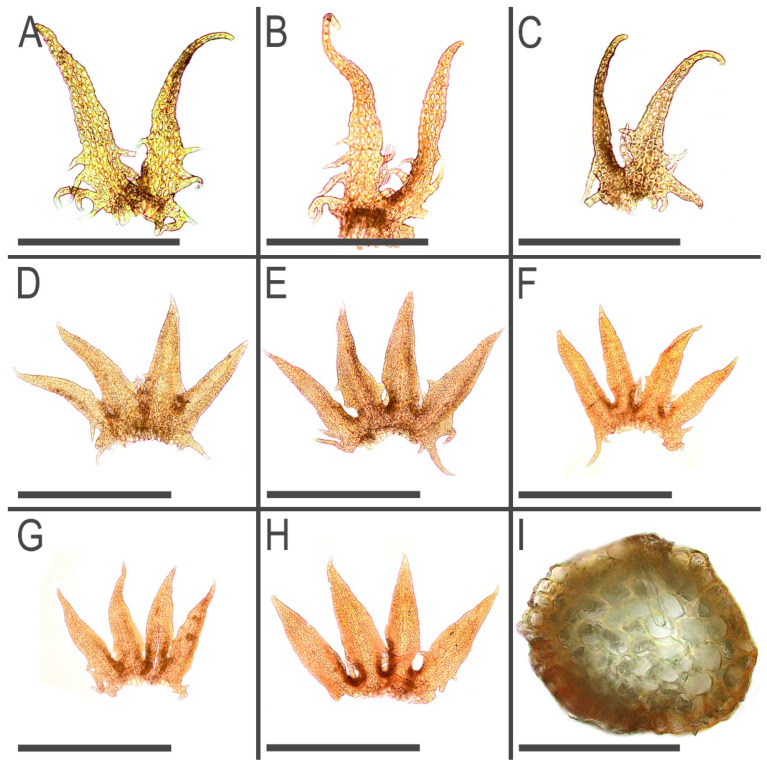
*Tetralophozia pusilla* (Steph.) Bakalin et Vilnet: **A**–**C**—underleaves; **D**–**H**—leaves; **I**—stem cross section. Scales: **A**–**C**—300 µm; **D**–**H**—500 µm; **I**—100 µm. All from J-88-40-15 (VBGI).

**Figure 12:**
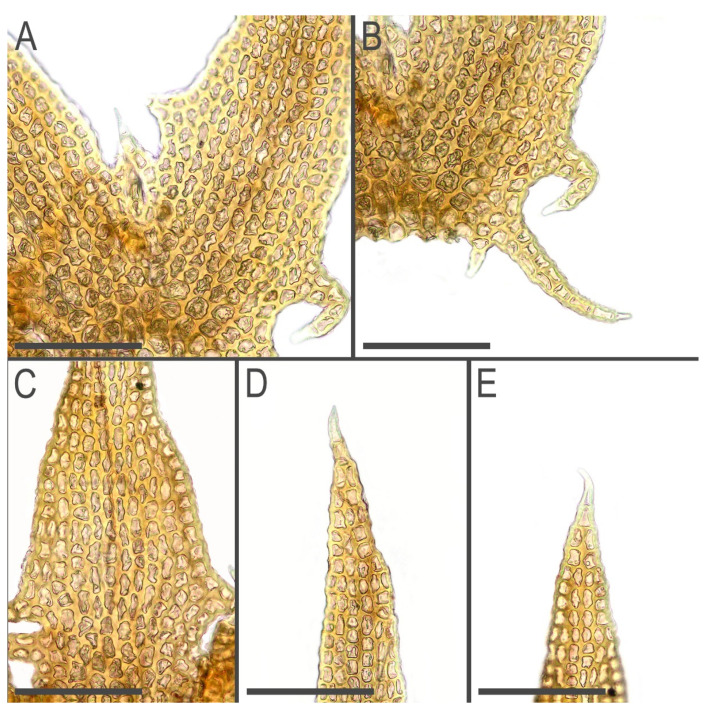
*Tetralophozia pusilla* (Steph.) Bakalin et Vilnet: **A**–**C**—leaf lobe bases; **D**,**E**—leaf lobe apex. Scales: **A**–**E**—100 µm. All from J-88-40-15 (VBGI).

**Figure 13:**
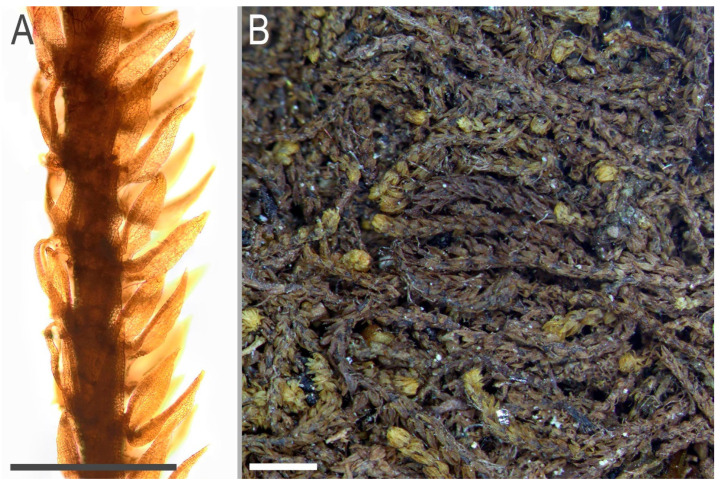
*Tetralophozia pusilla* (Steph.) Bakalin et Vilnet: **A**—plant habit. fragment. lateral view; **B**—mat. dorsal view. Scales: **A**—500 µm. **B**—2 mm. All from G00283395.

**Figure 14:**
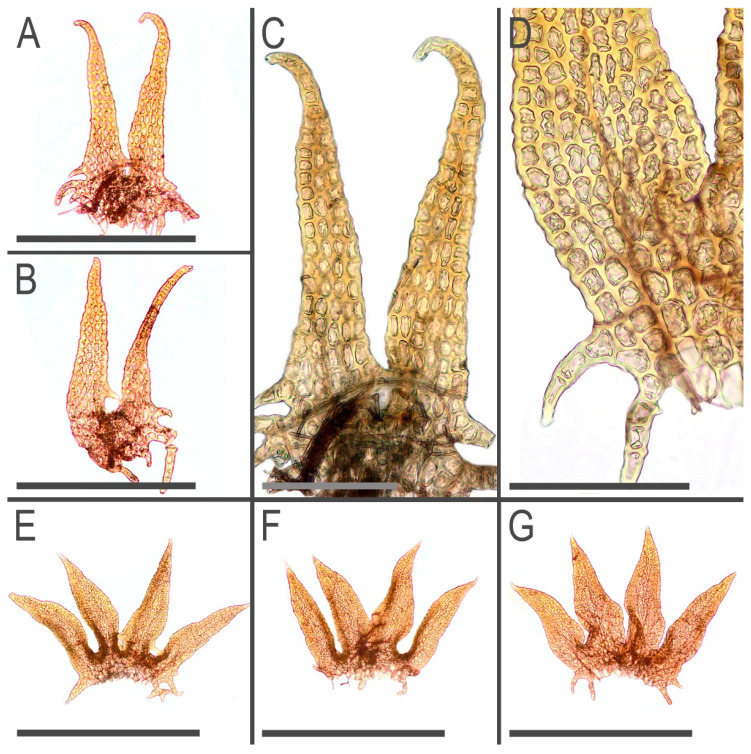
*Tetralophozia pusilla* (Steph.) Bakalin et Vilnet: **A**–**C**—underleaves; **D**—leaf lateral lobe base; **E**–**G**—leaves. Scales: **A**,**B**—300 µm. **C**,**D**—100 µm; **E**–**G**—500 µm. All from G00283395 (type).

Description (based on specimens examined placed in the Table 5). Plants brown to yellowish brown, with yellowish apices in herbarium, when fresh, commonly green-brownish and with greenish yellowish shoot apices, ascending, never distinctly creeping or erect, forming very loose mats, commonly with admixture of some pleurocarpous mosses, 10–20 µm long and 0.4–0.6 mm wide (dry plants 0.2–0.4 mm wide), shoots look loosely vermiform. Rhizoids virtually absent to sparse, 500–800 µm long, grayish to brown, erect or in obliquely spreading fascicles, originating from the stem near underleaf bases. Branching sparse, terminal, *Frullania*-type (looking as dichotomous) and as subfloral innovations (1–)2 per gynoecium; stem cross section slightly transversely elliptic, 115–125 × 150–175 µm, with loosely defined cortex, distinctly striolate, outer cells with thick walls, with nearly rounded lumens, ca. 7–10 µm in diameter, inner cells 8–15 µm in diameter, with thin walls and large, mostly concave trigones. Leaves transversely inserted, mainly subimbricate or (rarely, in weaker shoots only) obliquely spreading; subimbricate leaves distinctly concave, with lower 1/4–1/3 erect spreading, then suddenly curved and going subparallel to the stem; (3–)4-lobed, lobes subequal (weak plants have predominantly 3-lobed leaves, with dorsal lobe larger), 400–500 µm long and (at the level of leaf lamina) 250–450 µm wide, undivided part 50–90 µm (3–6 cells) high, lobe apices prominently acuminate, with straight axis and 1–3-celled uniseriate ends, sinus strongly recurved in the base, each lobe with 1–3 acute 1–6-celled teeth in lower third or basal teeth completely absent (as in the type specimen), each lateral side of the leaf have one more additional, commonly curved and sometimes branched, long (to 12 cells long) tooth. Underleaves bilobed, 300–400 µm long, 120–150 µm wide (at the level of lamina), undivided part 30–50 µm (2–3 cells) high, lobes prominently acuminate with straight or distinctly curved axes and 3–4-celled uniseriate ends, sinus recurved in the base, lobe bases in the sinus with 2–4 teeth, lateral underleaf bases with 3–5 teeth, the basal tooth the largest, sometimes branched and strongly curved, 1–2 basal teeth sometimes terminating with slime papilla. Cells in the lobe base 10–20 × 10–12 µm, oblong to nearly isodiameteric, strongly vermiculately thickened, with prominent, large, and convex trigones; cuticle papillose, sometimes scarcely so. Generative structures unknown (Figure 11, Figure 12, Figure 13 and Figure 14).

Comment. The same written in the comment about papillae on the leaf and stem surface under *T. filiformis* should be applied to the type of *C. pusillus*. The papillae are difficult to observe and the surface sometimes looks virtually smooth. This may be explained by the indenting of surface elements inward and also because papillae in fresh material even are not prominently high in this species.

## 5. Materials and Methods

### 5.1. The Tetralophozia Overview

**Figure 15:**
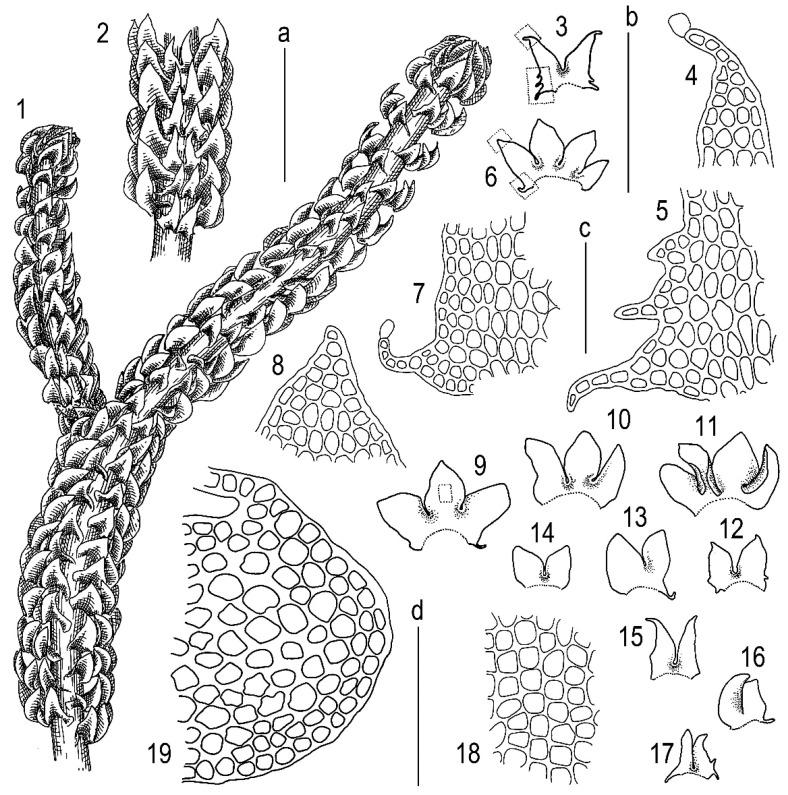
*Tetralophozia setiformis* (Ehrh.) Schljakov (somewhat depauperate plants): **1**—plant habit. dorsal view; **2**—plant habit. fragment. ventral view; **3**,**12**–**17**—underleaves; **4**—underleaf lobe apex; **5**—underleaf base (from 3); **6**,**9**–**11**—leaves; **7**—leaf lateral base (from 6); **8**—leaf lobe apex; **18**—cells in lobe base; **19**—stem cross section. fragment. Scales: **a**—1 mm. for 1. 2; **b**—1 mm. for 3,6,9–17; **c**—100 µm. for 4,5,7,8; **d**—100 µm. for 18,19. All from Prim-81-3-17 (VBGI).

The genus *Tetralophozia* started to be widely accepted in Europe from Schljakov [14], who first made the new combinations to raise *Chandonanthus* subg. *Tetralophozia* R.M. Schust. into the genus and transferred *Chandonanthus setiformis* into *Tetralophozia*. *Chandonanthus* subg. *Tetralophozia* R.M. Schust. was described 16 years prior to Schljakov’s new combination [15]. Seven years after Schljakov [14], Urmi [2] transferred *Chandonanthus filiformis* to *Tetralophozia*, Váňa [16] did the same with *Blepharostomum cavallii* Gola and Schuster [17]—with *Chandonanthus piliferus* Steph. The genus *Tetralophozia*, as accepted by Söderström et al. [18], includes 4 species worldwide. *Tetralophozia setiformis* generally has an arctic-alpine circumpolar distribution. The species is widely spreading southward in the Holarctic by the mountain ranges in Europe (Alps, Carpathians) but far less so in North America and Asia, where the southernmost localities lie at 43° N in the Russian Far East mainland. The species is also illustrated here (Figure 15), based on the somewhat depauperate plants from one of the southernmost localities (43° N) in Asian mainland in Primorsky Territory. The species is almost exclusively epilithic or grows on humus in cliff crevices, as exclusion in some sites in Northeast Asia may occur on lying branches of *Pinus pumila*, a dwarf shrubby pine. The *Tetralophozia filiformis* complex, discussed in the present paper in detail, has (as one, although complex unit) an oro-temperate-oro-boreal amphioceanic range, and all records are from rocky substrates. *Tetralophozia cavalli* (Gola) Váňa is confined to the Central African high mountains (Ruwenzori Mt., Virunga Mts., Kilimanjaro Mt.). The species occurs on the bark of trees from middle to high elevations [16]. *Tetralophozia pilifera* (Steph.) R.M. Schust. is a New Guinean endemic species growing mostly in epiphytic habitats and occurring much more rarely in epixylous habitats [19]. All recognized taxa of the genus are quite variable morphologically, although there have been no attempts to confirm whether this is indeed an environmentally induced variation that does not correlate with molecular-genetic features.

As it was admitted starting from Urmi [2], *Chandonanthus pusillus* Steph. was treated as the synonym of *Tetralophozia filiformis*, although Schuster [20] called this synonymy ‘presumable’ and noted “*T. filiformis* is more strongly armed with spinescent teeth and cilia, which may occur far up the lobe margins; in *C. pusillus*, lobes are entire or bear 1–2 small teeth just above their bases”. The type localities of *Chandonanthus filiformis* and *C. pusillus* are, although in East Asia, situated at a strong distance from one another. The type locality of *Chandonanthus filiformis* is in Ma’ershan (‘Ma Cul Chan’ by Delavay), which is in Yunnan Province of China ([1]; G00112121!). The locality is situated within the small Ma’ershan floristic province belonging to the Jinsha River floristic Subregion. This small province houses 19 stenochoric endemic vascular plants, which is quite a lot among 84 floristic provinces within the administrative Yunnan Province of China [21]. The type locality of *Chandonanthus pusillus* is in Komagadake Mt. in Yamanashi Prefecture ([1]; G00283305!). The latter is situated within the Southern Japanese Alps [=Minami Alps]—a quite distinctive area due to high level of taxonomic diversity as it was recognized by United Nations Educational, Scientific and Cultural Organization (UNESCO) (http://www.unesco.org/new/en/natural-sciences/environment/ecological-sciences/biosphere-reserves/asia-and-the-pacific/japan/minami-alps, accessed on 18 May 2021). The bryofloristic richness of this region is also obvious [22,23].

### 5.2. Specimens

The studied specimens of the *Tetralophozia filiformis* complex that are kept in VBGI, JNU, and KPABG were collected by the authors or other collectors. Types were loaned from G (two types: *Chandonanthus pusillus* and *C. filiformis*). Where possible, the specimens were processed via molecular-genetic analysis. The basic principle that was accepted in this work was not only to study quite old-type materials (that are also almost always impossible to sequence) but also to try to collect specimens in type localities or nearby—to reveal their real morphological variability in classic localities and to obtain material suitable for molecular-genetic analysis. Therefore, we visited Komagadake Mt. in Japan and the Ma’ershan area (the position of the type locality is quite indefinite) in Yunnan Province of China. In addition, other areas in southern China (Sichuan Province), the Korean Peninsula and Russian Siberia were explored. In total, 17 specimens (both collected by us, other collectors and requested from other herbaria) were studied: 4 from Russian Siberia, 1 from the Russian Far East, 6 from the Korean Peninsula, 1 from Japan, 1 from North Vietnam and 3 from China. Additionally, three specimens of *T. setiformis* were included for molecular-genetic comparison. All specimens are listed in Table 5, along with the GenBank accession numbers.

### 5.3. Molecular-Genetic Study

The monophyly of the genus *Tetralophozia* remains questionable since two species from the genus *Plicanthus* R.M. Schust. were subsequently found within it [8,24]. Due to the absence of suitable sampling of *Plicanthus*, we were not able to clarify the generic concept of both genera and concentrated here only on affinity among known *Tetralophozia* species. For molecular estimation, we selected 11 specimens of *Tetralophozia* and 11 species from the family Anastrophyllaceae, and *Lophozia ascendens* (Warnst.) R.M. Schust. from Lophoziaceae was chosen as an outgroup. In total, ITS1–2 nrDNA and *trn*L–F cpDNA sequence data for 14 samples were taken from our previous studies, GenBank accession numbers for them are provided in Figure 1.

DNA was extracted with a DNeasy Plant Mini Kit (Qiagen, Hilden, Germany). The primers suggested by White et al. [25] for ITS1–2, Taberlet et al. [26] for *trn*L–F and Shaw et al. [27] for *trn*G-intron were used for amplification and sequencing. Polymerase chain reaction (PCR) was carried out in 20 µL volumes with the following protocol: 3 min at 94 °C, 30 cycles (30 s 94 °C, 40 s 56 °C for ITS1–2 and *trn*L–F or 64 °C for *trn*G-intron, 60 s 72 °C), 2 min of final extension at 72 °C. Amplified fragments were visualized on 1% agarose TAE gels by EthBr staining, purified using the QIAquick Gel Extraction Kit (Qiagen, Germany), and used as a template in sequencing reactions with the ABI Prism BigDye Terminator v. 3.1 Ready Reaction Kit (Applied Biosystems, Waltham, MA, USA) following the standard protocol provided for the 3730 DNA Analyser (Applied Biosystems, Waltham, MA, USA).

Newly generated sequences were assembled and aligned with previously obtained sequences in BioEdit 7.0.1 [28]. Alignments for ITS1–2 and *trn*L–F were produced manually; all positions were considered. Due to the absence of appropriate data, the *trn*G-intron dataset was not produced. Preliminary phylogenetic estimation revealed congruent results from both datasets; thus, they were combined in a single dataset ITS1–2+*trn*L–F for subsequent analyses by the maximum parsimony (MP) method with TNT v.1.5 [29] and the maximum likelihood (ML) method with PhyML v.3.0 [30]. The MP analysis involved a New Technology Search for the minimal length tree by five iterations and 1000 bootstrap replicates, and default settings were used for other parameters. The software ModelGenerator [31] selected the best-fit evolutionary model of nucleotide substitutions, namely, TN+I+G. The stopping frequency criterion for bootstrapping suggested 450 replicates as enough to reach BS convergence with Pearson average ρ100 = 0.997020 realized in RAxML v7.2.6 [32]. Thus, ML analysis was performed with the TN+I+G model, 500 bootstrap replicates and gamma distribution of the rate heterogeneity among sites with four rate categories.

The average pairwise *p*-distances for the genus *Tetralophozia* were calculated in Mega 11 [33] based on each DNA locus using the pairwise deletion option for counting gaps.

### 5.4. Climate Analysis

Since the climate is obviously changing in the distribution range of the *Tetralophozia filiformis* complex, we obtained the bioclimate variables for collecting localities of the specimens: 1) studied by molecular and/or morphological methods, and 2) randomly selected from the GBIF database (https://www.gbif.org/ru/occurrence/search?taxon_key=2689448, accessed on 18 May 2021) and for two specimens from Spain on which the first report of the taxon for Europe was based [2]. In total, 22 localities were selected, and 19 bioclimate variables were identified based on information provided in WorldClim software (https://www.worldclim.org/): BIO1 = Annual Mean Temperature, BIO2 = Mean Diurnal Range (Mean of monthly (max temp − min temp))m BIO3 = Isothermality (BIO2/BIO7) (×100), BIO4 = Temperature Seasonality (standard deviation ×100), BIO5 = Max Temperature of Warmest Month, BIO6 = Min Temperature of Coldest Month, BIO7 = Temperature Annual Range (BIO5–BIO6), BIO8 = Mean Temperature of Wettest Quarter, BIO9 = Mean Temperature of Driest Quarter, BIO10 = Mean Temperature of Warmest Quarter, BIO11 = Mean Temperature of Coldest Quarter, BIO12 = Annual Precipitation, BIO13 = Precipitation of Wettest Month, BIO14 = Precipitation of Driest Month, BIO15 = Precipitation Seasonality (Coefficient of Variation), BIO16 = Precipitation of Wettest Quarter, BIO17 = Precipitation of Driest Quarter, BIO18 = Precipitation of Warmest Quarter, BIO19 = Precipitation of Coldest Quarter. The obtained data were then tested using multivariate analysis (using Past ver. 4.03c [34]). The hierarchical clustering was based on Ward’s method [35], and Euclidean distance was used to check the results shown by detrended correspondence analysis (DCA). DCA was visualized in a three-dimensional grid graph, with the third dimension given by the color gradient.

## 6. Conclusions

The study illustrates that when only a few materials and from limited regions are available, the distinctive traits of the regional ‘populations’ may be overlooked, and a potentially new taxa could be neglected. In our case, when we had an experience restricted to the specimens collected in South Siberia, we certainly could not recognize that a taxon different from *Tetralophozia filiformis* is in hand because all our attention was attracted to the robust difference of collected material from *T. setiformis* (another locally known taxon). The involvement of additional material from type localities (including types) derived from other regions in East Asia revealed noticeable variation within widely treated *T. filiformis* in four main groups of species features: molecular genetics, morphology, ecology (including climate characteristics), and geography. The situation is somewhat similar to the recently published *Ptilidium himalayanum*, the species molecularly more different from the pair *P. ciliare*–*P. pulcherrimum* than the taxa constructing the pair and occupying a distinctly defined area in the Sino-Himalaya [36].

Treating *Tetralophozia sibirica* as the terminal link in the adaptation process to the cold and dry climate within *T. filiformis* s. lat. complex, then it could be stressed the main morphological pathways of this ‘adaptation’ (shortening leaf lobes, not so deeply divided leaves, sparser leaf dentation) seem imaginable in regard to the northerly distributed Arctic-alpine *T. setiformis* (showing the same features in much more pronounced manner). This regularity to possess shorter divided leaves northward may be compared with trends observed in other genera, e.g., (1) *Scapania* with deeply divided leaves are not present in Arctic-distributed taxa (in spite of generally high taxonomic diversity of this genus in ‘high’ latitudes), (2) the wide distribution of *Gymnomitrion* taxa with shallowly-lobed leaves in the Arctic and the absence taxa with deeply-divided leaves there.

## Figures and Tables

**Table 1:** The value of *p*-distances for the genus *Tetralophozia*.

No	Species	Infraspecific *p*-Distances, ITS1–2/*trn*L–F,%	Infrageneric *p*-Distances, ITS1–2/*trn*L–F,%		
			1	2	3
1	*T. sibirica*	0.3/0.7			
2	*T. filiformis*	1.1/0.0	2.5/1.2		
3	*T. pusilla*	0.3/0.2	2.7/1.0	1.3/0.2	
4	*T. setiformis*	0.5/0.1	3.3/2.7	2.9/1.3	3.2/1.6

**Table 2:** The bioclimate indices for each locality where the specimen of *Tetralophozia filiformis* s.l. was collected (regardless was specimen studied or not) *.

**№**	**Mark in Figure 2**	**Accepted Name**	**Field no.**	**Latitude**	**Longitude**	**BIO01**	**BIO02**	**BIO03**	**BIO04**	**BIO05**	**BIO06**	**BIO07**	**BIO08**	**BIO09**	
1	square	*T. filiformis*	C-39-1-17	29.977055	101.88477	4.071	11.008	37.065	695.248	19.500	−10.200	29.700	12.567	−4.417	
2	square	*T. filiformis*	C-40-16-17	29.976361	101.885194	4.071	11.008	37.065	695.248	19.500	−10.200	29.700	12.567	−4.417	
3	square	*T. filiformis*	C-73-44-18	26.59494	99.764333	7.371	9.108	36.727	487.924	19.900	−4.900	24.800	13.117	1.917	
4	square	*T. filiformis*	V-10-6-19	22.505861	103.587805	9.813	7.758	39.382	420.606	19.600	−0.100	19.700	14.717	5.017	
5	triangle	*T. pusila*	3735	35.664083	127.735583	9.108	10.767	29.660	923.811	26.700	−9.600	36.300	20.017	−2.733	
6	triangle	*T. pusila*	111058	35.333805	127.731972	5.796	8.608	26.487	867.395	21.900	−10.600	32.500	15.900	−2.683	
7	triangle	*T. pusilla*	Kor-23-27-15	35.33166	127.73416	7.400	9.183	27.744	869.190	23.800	−9.300	33.100	17.533	−1.200	
8	triangle	*T. pusilla*	Kor-25-1-15	35.43472	127.73083	11.188	10.958	29.941	929.507	29.400	−7.200	36.600	22.183	−0.667	
9	triangle	*T. pusilla*	Kor-27-20-15	35.325	127.70694	6.475	8.900	27.052	868.830	22.800	−10.100	32.900	16.583	−2.083	
10	triangle	*T. pusilla*	Kor-7-13-11	38.12777	128.44861	6.229	8.525	24.568	943.496	23.000	−11.700	34.700	17.000	−5.933	
11	triangle	*T. pusilla*	J-88-40-15	35.74556	138.23389	2.033	8.900	27.900	837.942	18.400	−13.500	31.900	12.300	−7.983	
12	circle	*T. sibirica*	MI-1077-97	52.06666	134.86666	−8.296	15.075	27.409	1491.022	18.300	−36.700	55.000	9.833	−26.683	
13	circle	*T. sibirica*	37-11-00	56.90694	120.052305	−7.938	12.008	22.279	1576.736	19.100	−34.800	53.900	11.467	−23.967	
14	circle	*T. sibirica*	13-24-01	51.42925	105.040583	−0.975	9.800	23.113	1210.906	19.500	−22.900	42.400	14.200	−14.100	
15	circle	*T. sibirica*	411	56.911388	117.80944	−9.108	12.483	24.193	1507.015	17.000	−34.600	51.600	10.050	−26.933	
16	circle	*T. sibirica*	C. C. Exsiccata. 411	56.9115	117.8095	−9.108	12.483	24.193	1507.015	17.000	−34.600	51.600	10.050	−26.933	
17	snowflake	*T. filiformis* s.l. (Spain)	(Urmi. 1983) Urmi2028 (not seen)	43.23194	−1.52222	12.017	8.183	38.601	467.412	22.300	1.100	21.200	7.667	17.667	
18	snowflake	*T. filiformis* s.l. (Spain)	(Urmi. 1983) Urmi 2220 (not seen)	43.23333	−1.53611	12.813	8.425	39.929	455.043	23.000	1.900	21.100	8.517	18.233	
19	snowflake	*T. filiformis* s.l.	GBIF ALA B43214 (Alaska)	56.160077	−131.9698	6.183	7.117	32.057	547.309	16.400	−5.800	22.200	3.083	11.350	
20	snowflake	*T. filiformis* s.l.	GBIF ALA B43238 (Alaska)	56.373755	−132.100001	6.013	6.825	30.199	562.980	16.400	−6.200	22.600	2.633	11.383	
21	snowflake	*T. filiformis* s.l.	GBIF UBC B228679 (Canada)	49.67	−123.16	9.100	7.183	32.652	546.884	19.500	−2.500	22.000	3.033	15.683	
22	snowflake	*T. filiformis* s.l.	GBIF E BGBASE: 686287 (Bhutan)	27.544167	90.722778	9.754	10.508	42.202	506.442	22.100	−2.800	24.900	15.467	4.117	
**№**	**Mark in Figure 2**	**Accepted Name**	**Field no.**	**Latitude**	**Longitude**	**BIO10**	**BIO11**	**BIO12**	**BIO13**	**BIO14**	**BIO15**	**BIO16**	**BIO17**	**BIO18**	**BIO19**
1	square	*T. filiformis*	C-39-1-17	29.977055	101.88477	12.567	−4.417	755.000	159.000	3.000	94.283	417.000	11.000	417.000	11.000
2	square	*T. filiformis*	C-40-16-17	29.976361	101.885194	12.567	−4.417	755.000	159.000	3.000	94.283	417.000	11.000	417.000	11.000
3	square	*T. filiformis*	C-73-44-18	26.59494	99.764333	13.117	1.150	868.000	147.000	13.000	62.081	394.000	57.000	394.000	64.000
4	square	*T. filiformis*	V-10-6-19	22.505861	103.587805	14.717	4.333	1756.000	367.000	5.000	87.810	967.000	40.000	967.000	71.000
5	triangle	*T. pusila*	3735	35.664083	127.735583	20.017	−2.733	1510.000	345.000	30.000	81.675	833.000	110.000	833.000	110.000
6	triangle	*T. pusila*	111058	35.333805	127.731972	15.900	−5.200	1833.000	402.000	36.000	77.820	987.000	135.000	987.000	139.000
7	triangle	*T. pusilla*	Kor-23-27-15	35.33166	127.73416	17.533	−3.650	1741.000	379.000	33.000	78.298	943.000	127.000	943.000	130.000
8	triangle	*T. pusilla*	Kor-25-1-15	35.43472	127.73083	22.183	−0.667	1401.000	311.000	24.000	82.863	779.000	94.000	779.000	94.000
9	triangle	*T. pusilla*	Kor-27-20-15	35.325	127.70694	16.583	−4.567	1784.000	392.000	35.000	78.138	963.000	132.000	963.000	134.000
10	triangle	*T. pusilla*	Kor-7-13-11	38.12777	128.44861	17.133	−5.933	1334.000	304.000	27.000	84.344	747.000	96.000	727.000	96.000
11	triangle	*T. pusilla*	J-88-40-15	35.74556	138.23389	12.300	−8.317	2066.000	325.000	46.000	58.026	900.000	167.000	900.000	239.000
12	circle	*T. sibirica*	MI-1077-97	52.06666	134.86666	9.833	−26.683	820.000	171.000	9.000	84.076	446.000	34.000	446.000	34.000
13	circle	*T. sibirica*	37-11-00	56.90694	120.052305	11.467	−27.150	478.000	106.000	5.000	93.877	288.000	19.000	288.000	20.000
14	circle	*T. sibirica*	13-24-01	51.42925	105.040583	14.200	−15.733	507.000	120.000	7.000	89.071	294.000	26.000	294.000	28.000
15	circle	*T. sibirica*	411	56.911388	117.80944	10.050	−26.933	442.000	98.000	4.000	95.500	270.000	14.000	270.000	14.000
16	circle	*T. sibirica*	C. C. Exsiccata. 411	56.9115	117.8095	10.050	−26.933	442.000	98.000	4.000	95.500	270.000	14.000	270.000	14.000
17	snowflake	*T. filiformis* s.l. (Spain)	(Urmi. 1983) Urmi 2028 (not seen)	43.23194	−1.52222	18.067	6.733	1241.000	134.000	66.000	21.016	377.000	226.000	233.000	348.000
18	snowflake	*T. filiformis* s.l. (Spain)	(Urmi. 1983) Urmi 2220 (not seen)	43.23333	−1.53611	18.667	7.600	1232.000	136.000	65.000	21.606	377.000	223.000	235.000	344.000
19	snowflake	*T. filiformis* s.l.	GBIF ALA B43214 (Alaska)	56.160077	−131.9698	12.967	−0.283	2914.000	440.000	137.000	36.545	1071.000	444.000	470.000	832.000
20	snowflake	*T. filiformis* s.l.	GBIF ALA B43238 (Alaska)	56.373755	−132.100001	12.967	−0.717	2749.000	424.000	130.000	37.476	1016.000	421.000	447.000	780.000
21	snowflake	*T. filiformis* s.l.	GBIF UBC B228679 (Canada)	49.67	−123.16	15.700	2.383	2692.000	470.000	68.000	60.630	1223.000	234.000	239.000	996.000
22	snowflake	*T. filiformis* s.l.	GBIF E BGBASE: 686287 (Bhutan)	27.544167	90.722778	15.467	3.150	974.000	211.000	5.000	87.628	537.000	21.000	537.000	24.000

* BIO1 = Annual Mean Temperature; BIO2 = Mean Diurnal Range (Mean of monthly (max temp − min temp)); BIO3 = Isothermality (BIO2/BIO7) (×100); BIO4 = Temperature Seasonality (standard deviation ×100); BIO5 = Max Temperature of Warmest Month; BIO6 = Min Temperature of Coldest Month; BIO7 = Temperature Annual Range (BIO5–BIO6); BIO8 = Mean Temperature of Wettest Quarter; BIO9 = Mean Temperature of Driest Quarter; BIO10 = Mean Temperature of Warmest Quarter; BIO11 = Mean Temperature of Coldest Quarter; BIO12 = Annual Precipitation; BIO13 = Precipitation of Wettest Month; BIO14 = Precipitation of Driest Month; BIO15 = Precipitation Seasonality (Coefficient of Variation); BIO16 = Precipitation of Wettest Quarter; BIO17 = Precipitation of Driest Quarter; BIO18 = Precipitation of Warmest Quarter; BIO19 = Precipitation of Coldest Quarter.

**Table 3:** Normalized values of DCA for each compared flora. in accordance to the specimen numbers in the Table 2.

No	DCA		
	Axis 1 (X)	Axis 2 (Y)	Axis 3 (Z)
1	46.00	24.00	13.00
2	46.00	24.00	13.00
3	68.00	21.00	6.00
4	82.00	34.00	6.00
5	68.00	18.00	3.00
6	76.00	18.00	1.00
7	74.00	18.00	1.00
8	65.00	18.00	4.00
9	75.00	18.00	1.00
10	64.00	16.00	4.00
11	84.00	13.00	0.00
12	25.00	5.00	14.00
13	1.00	0.00	19.00
14	15.00	5.00	17.00
15	0.00	0.00	20.00
16	0.00	0.00	20.00
17	109.00	5.00	0.00
18	109.00	7.00	0.00
19	124.00	7.00	3.00
20	123.00	6.00	3.00
21	126.00	25.00	30.00
22	64.00	32.00	10.00

**Table 4:** The correlation between values of each bioclimate and values obtained in the axis.

Bioclimate Indices	Axis 1 (X)	Axis 2 (Y)	Axis 3 (Z)
BIO01	0.803697855	0.610695077	−0.577801558
BIO02	−0.843076109	−0.290005672	0.423780285
BIO03	0.505493332	0.586507785	−0.228884513
BIO04	−0.843868158	−0.616066578	0.463700903
BIO05	0.14350037	0.347252607	−0.4291601
BIO06	0.849729356	0.6223567	−0.536279158
BIO07	−0.873734551	−0.569422916	0.452828573
BIO08	−0.348310994	0.390696387	−0.310065311
BIO09	0.92838766	0.418628246	−0.450694567
BIO10	0.475267305	0.344690362	−0.51527616
BIO11	0.846435514	0.614736776	−0.540815429
BIO12	0.858486569	0.221188912	−0.359161055
BIO13	0.689773053	0.400419913	−0.330532825
BIO14	0.795660002	−0.224500064	−0.359993131
BIO15	−0.79457789	0.224928435	0.473925358
BIO16	0.703195812	0.421098664	−0.315688842
BIO17	0.821092389	−0.195327474	−0.390334625
BIO18	0.189870135	0.48707667	−0.596398852
BIO19	0.778554616	−0.068321891	0.013610967

**Table 5:** Specimens examined (excluding the types of *Tetralophozia filiformis* (Steph.) Urmi and *T. pusilla* (Steph.) Bakalin et Vilnet).

No	Name	Label Data	Latitude. N	Longitude. E	Field Number Plus Barcode and Herbarium Acronym (in Brackets)	GenBank Accession Number	
						ITS1–2 nrDNA	*trn*L–F/*trn*G–intron cpDNA
1	*Tetralophozia filiformis*	China. Sichuan Province. Bakalin & Klimova. 13 October 2017	29.977055	101.88477	China-39-1-17 (VBGI- 37281. KPABG-122599 duplicate)	MZ231275	MZ229433/-
2	*T. filiformis*	China. Sichuan Province. Bakalin & Klimova. 13 October 2017	29.976361	101.885194	China-40-16-17 (VBGI-37325)	no data	no data
3	*T. filiformis*	China. Yunnan Province. Bakalin & Ma. 11 October 2018 near locus classicus of the species	26.59494	99.764333	C-73-44-18 (VBGI)	MZ231276	MZ229434/-
4	*T. filiformis*	Vietnam. Lai Châu Province. Bakalin & Klimova	22.505861	103.587805	V-10-6-19 (VBGI-65792)	no data	no data
5	*T. pusilla*	Republic of Korea Gyeongsang-do. Choi. 14 June 2009	35.664083	127.735583	Choi-3735 (JNU. duplicate VBGI)	no data	no data
6	*T. pusilla*	Republic of Korea Gyeongsang-do. Choi. 1 October 2011	35.333805	127.731972	Choi-111058 (JNU. duplicate VBGI)	no data	no data
7	*T. pusilla*	Republic of Korea. Gyeongsangnam-do. Bakalin. 5 May 2015	35.33166	127.73416	Kor-23-27-15 (VBGI)	no data	no data
8	*T. pusilla*	Republic of Korea. Gyeongsangnam-do. Bakalin. 6 May 2015	35.43472	127.73083	Kor-25-1-15 (VBGI)	no data	no data
9	*T. pusilla*	Republic of Korea. Gyeongsangnam-do. Bakalin 7 May 2015	35.32500	127.70694	Kor-27-20-15 (VBGI). (KPABG-120508. duplicate)	MZ231277	MZ229435/-
10	*T. pusilla*	Republic of Korea. Gangwon-do. Bakalin. 11 May 2015	38.12777	128.44861	Kor-7-13-11 (VBGI)	no data	no data
11	*T. pusilla*	Japan. Yamanashi Prefecture. Bakalin. 1 October 2015 near locus classicus of the species	35.74556	138.23389	J-88-40-15 (VBGI-5796. KPABG-123441 duplicate)	MZ231278	MZ229436/MZ229442
12	*T. sibirica*	Russia. Khabarovsk Territory. Ignatov. 15 August 1997	52.06666	134.86666	MI-1077-97 (KPABG-116740)	no data	MZ229438/-
13	*T. sibirica*	Russia. Amurskaya Province. Bakalin. 17 August 2000	56.90694	120.052305	37-11-00 (KPABG-101730)	no data	MZ229439/-
14	*T. sibirica*	Russia. Buryatia Republic. Konstantinova. 4 August 2001	51.42925	105.040583	13-24-01 (KPABG-102424)	EU791792	EU791669/-
15	*T. sibirica*	Russia. Zabaikalsky Territory. Mamontov. 7 July 2013	56.911388	117.80944	411 (KPABG-121349)	MZ231279	MZ229437/-
16	*T. setiformis*	Russia: Buryatia Republic. Konstantinova & Savchenko. 8 August 2002	51.185591	105.181264	123-2-02 (KPABG-121659)	EU791793	EU791670/-
17	*T. setiformis*	Russia: Kamchatka Territory. Bakalin. 13 July 2006	55.901388	158.782777	99-06 (KPABG-112052)	MZ231281	MZ229441/-
18	*T. setiformis*	Russia: Murmansk Province. Konstantinova. 7 July 2007	67.3229	35.1623	K201-1-07 (KPABG-18022)	MZ231280	MZ229440/-

## Data Availability

Not applicable.
